# Retrospective, single center evaluation of transperineal prostate biopsy omitting antibiotic prophylaxis and omitting periinterventional screening for bacteriuria

**DOI:** 10.1007/s00345-025-05672-8

**Published:** 2025-06-19

**Authors:** Francois Leboutte, Tim Schykowski, Jeroen Van de Plas, Oscar Lemmer, Andreas Neisius

**Affiliations:** 1 Department of Urology, Krankenhaus der Barmherzigen Brüder Trier, Medical Campus Trier of the Johannes Gutenberg-University Mainz, Trier, Germany; 2 Department of Urology, Helios Dr. Horst Schmidt Kliniken Wiesbaden, Wiesbaden, Germany

**Keywords:** Prostatic neoplasms [MeSH], Image-guided biopsy [MeSH], Transperineal, Transrectal, Fusion biopsy, Intraoperative complications [MeSH]

## Abstract

**Purpose:**

Although the pursuit of effective and safe early detection methods for prostate cancer has led to advancements in non-invasive tools, there are approximately one million prostate biopsies per year performed annually in the EU [11]. The use of the transperineal approach to prostate biopsy is increasing because it offers a significantly reduced incidence of post-biopsy sepsis complications compared to transrectal biopsies and therefore is the recommended approach in the European Association of Urology guidelines. However, the consensus on the standard of care for antibiotic prophylaxis in some or all transperineal biopsy cases is only beginning to be established. As with transrectal biopsies, there are concerns about antibiotic stewardship, antibiotic side effects and labor and material costs associated with prophylaxis.

**Methods:**

This retrospective study analyzed 636 patients who underwent transperineal prostate biopsies without the use of antibiotic prophylaxis between January 2019 and August 2020. The primary endpoint was the rate of postinterventional infectious complications, with secondary endpoints including the rate of general complications and associated risk factors.

**Results:**

The rate of all complications was 1.9%. There were 7/636 (1.1%) infectious complications, of which 3 (0.50%) were prostatitis, 1 (0.16%) was epididymitis, 2 (0.3%) were infections resulting in hospitalization and 1 (0.16%) urosepsis with ICU care. No identified risk factors were associated with infectious complications or post-interventional bleeding. Notably, the cohort was not systematically screened for bacteriuria before biopsy, and patients usually categorized as high risk for post-biopsy complications were not excluded. The rates of infectious complications and sepsis were lower than that reported for transrectal biopsies with antibiotic prophylaxis.

**Conclusion:**

This study supports the relative safety of omitting antibiotic prophylaxis in transperineal prostate biopsies, showcasing a minimal infectious complication rate. The findings contribute to the ongoing discourse on antibiotic stewardship, emphasizing the need for judicious use to mitigate resistance, avoid allergic side effects and decrease the labor and material costs associated with transperineal prostate biopsy.

## Introduction

The field of early prostate cancer detection has undergone substantial investigation to avoid unnecessary prostate biopsies, obtain accurate histopathological results and minimize morbidity. However, despite advances in non-invasive tools such as risk calculators, mpMRI, ultrasound and PSMA PET scan to determine the risk of prostate cancer, it is estimated that approximately one million prostate biopsies are performed annually in the European Union [11].

Because of fewer sepsis complications with use of the transperineal approach, EAU guidelines state that “The available evidence demonstrates that the transrectal approach should be abandoned in favour of the transperineal approach despite any possible logistical challenges”. It has been well established that antibiotic prophylaxis is necessary for the transrectal approach [6], likely due to fecal contamination of the biopsy needle as it passes through the rectal mucosa. However, the role of antibiotic prophylaxis in transperineal biopsies has been assessed over the last few years in many transperineal biopsy trials yet remains unclear [5, 9, 12–15].

However, a metanalysis by Basourakos [1] showed a small, statistically significant lower risk of infectious complications with antibiotic prophylaxis while another metanalysis by Castellani [4] found no significant difference between groups using or omitting antibiotic prophylaxis in rates of infection, fever, sepsis or readmission.

The cavalier use of antibiotics coupled with growing rates of bacterial resistance have become a significant public health issue. A review of antibiotic prescribing in hospitals by Hulscher et al. stated that assessments have found that up to 50% of hospital antibiotic use is inappropriate in 20–50% of cases [7]. Correct use of antibiotics is crucial for allowing continued effective control of bacterial infection. If omitting antibiotic prophylaxis in a widely used procedure such as prostate biopsy is safe, its impact on public health could be of enormous importance [2, 3].

In our study, we retrospectively analyzed the safety of omitting antibiotic prophylaxis in transperineal prostate biopsy without periinterventional screening for bacteriuria. The primary endpoint is rate of postinterventional infectious complications. The secondary endpoint is general complication rate and associated risk factors.

## Materials and methods

### Population

We retrospectively analyzed 650 consecutive patients who received transperineal prostate biopsies in our urology clinic between January 2019 and August 2020.

**Fig. 1:**
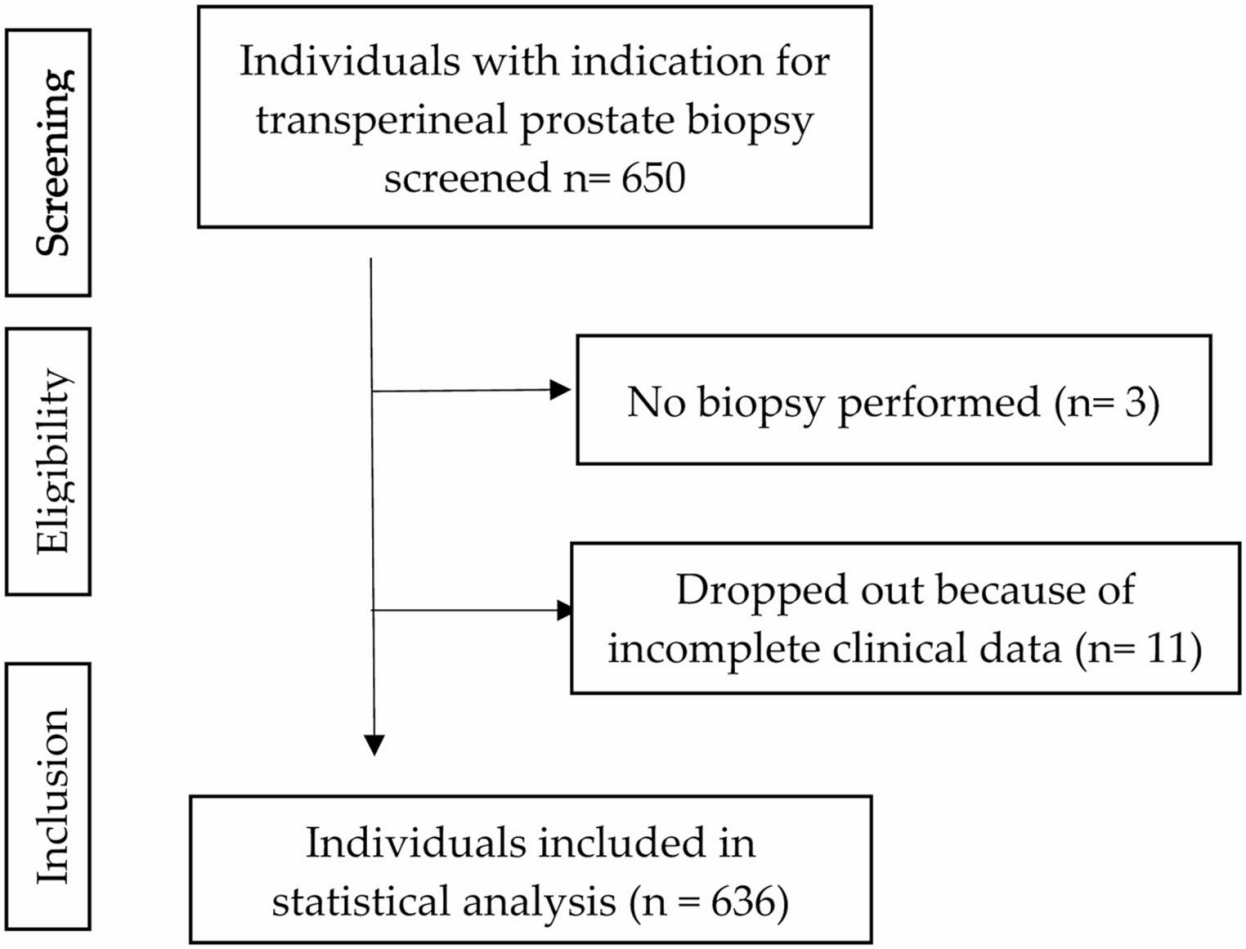
Study flow diagram

Inclusion criteria were: suspicion of prostate cancer, pathological PSA value/dynamics, pathological DRE, and/or pathological MRI Cases with inadequate documentation were excluded. Of the 650 patients, 636 met this inclusion criteria (Fig. 1).

### Biopsy procedure

Transperineal prostate biopsies were performed as outpatient procedures. Before completing the procedure, a short history was obtained, particularly asking about any prior operations of the genitourinary tract or history of indwelling catheters, family history, and existing diseases. A biopsy was not performed if the patient was taking anticoagulant medication, except for aspirin. Platelet aggregation inhibitors such as clopidogrel, ticagrelor etc. had to be paused for 7 days prior to the biopsy procedure. After excluding contraindications for prostate biopsy, general consent was obtained. No blood or urine samples were taken before the intervention.

The procedure was performed in lithotomy. The scrotum and penis were taped to the suprapubic area (unsterile Leukoplast– Hypafix 15 cm x 20 m, Beiersdorf AG, Hamburg, Germany). The perineum was shaved when necessary, and gross dirt particles were removed. Antiseptic disinfection was completed with 7.5% Povidone-Iodine solution (Braunol 7.5% Povidon Iod, Braun, Germany). Two dedicated urologists performed the transperineal biopsies. Local anesthesia of the skin and periprostatic.

blockade were performed with a *20* gauge needle (Sterican 20 G 0.9 × 70 mm, Braun, Germany) and 20 ml of Mepivicain 10 mg/ml (Mecain, Eugia Pharma PUREN, Malta). Periprostatic block was achieved by finger guidance with the injection of mepivicain below the prostatic apex, bilaterally in the levator ani, and along the needle’s path. 5 to 10 min were given for the optimal effect of local anesthesia. Biopsies were performed with a disposable template grid (Template Grid 17/18GA, Civco, Iowa, United States of America). A 24 cm long 18-gauge needle with a hand fire system (CORAZOR, UROMED, Germany) was used for sample collection.

Systematic biopsies were performed under local anesthesia (periprostatic nerve block). In MRI Fusion biopsy procedures, we added sedation to the local anesthesia (midazolam and ketamine) to minimize the risk of movement during the procedure.

UroNav Philips Invivo (Amsterdam, Netherlands) with a template grid was utilized for fusion biopsy combined with the BK 3000, BK Medical (Nærum, Denmark) rectal ultrasound. Systematic biopsies were performed with five cores per half gland. MRI fusion biopsies complemented systematic biopsies with 3 to 4 biopsies per lesion, depending on lesion size and counts.

After checking for gross hematuria and the ability to void, the patient was dismissed.

### Outcome variables

We classified the cases according to the following categorical outcome variables: previous biopsy, under active surveillance, previous prostatitis, diabetes mellitus, diabetes mellitus medication, obesity (cut-off for body mass index > 35), immunosuppression, anticoagulant therapy (aspirin, clopidogrel, phenprocoumon, or a direct oral anticoagulant), indwelling catheter, previous surgery, DRE, performing urologist, type of anesthesia, ISUP grade group, type of complication. In addition, we chose the following continuous outcome variables: age (years), prostate volume (cc), number of MRI lesions, number of cores, and the number of positive cores.

### Statistical methods

Statistical analyses were conducted using R (Version 4.0.5 for Windows). Non-parametric statistics were applied. Data were presented as raw values and percentages and medians with lower (Q1) and upper quartiles (Q3). We checked for relations between categorical data (prostate biopsy technique, prostate cancer detection) using chi-square test of independence. Correlation between PIRADS lesions and histological results (ISUP) of the biopsy was checked with the Spearman´s correlation test. We checked for risk factors using Generalized Linear Model (GLM). The threshold for a statistically significant difference was *p* < 0.05.

### Data source

We retrospectively obtained data from medical charts (i.e., categorical, and continuous outcome variables) between January 2022 and December 2022.

### Bias

We encountered two types of bias:

- Selection bias– the retrospective nature of the trial.
- Follow-up bias: follow-up data were collected by chart review from charts of our clinic. Outpatient complication treatment (i.e., prescription of antibiotics in the postinterventional course) by another physician may have occurred without being noted in our chart reviews.

## Results

Overall, 636 patients (median age 68 (62,73) years) with prostate cancer suspicion were included in this study. Demographics and biopsy results are highlighted in Tables 1 and 2, and 3. Of the 255 systematic biopsies performed, 125 cases of prostate cancer were confirmed histologically. In comparison, 238 cases of prostate cancer were confirmed histologically out of 378 fusion biopsies. Prostate cancer was significantly more frequently detected using fusion biopsies (*p* < 0.001). High-grade prostate cancer (ISUP > 3) was detected in 29.4% of systematic biopsies and 15.1% of fusion biopsies. This difference was statistically significant (*p* < 0.0001).

**Table 1:** Patient demographics and biopsy results

Age (years)	Missing	68 [62, 73] 0
PSA (ng/ml)	Missing	8.5 [6, 13.7] 9 (1.4%)
Prostate Volume (ccm)		52.0 [39, 72.3]
	Missing	8 (1.3%)
Indwelling catheter	No	598 (94.0%)
	Yes Missing	32 (5.0%) 6 (0.9%)
Active Surveillance		
	No	601 (94.5%)
	Yes Missing	29 (4.6%) 6 (0.9%)
Transperineal Biopsy technique		
	MRI Fusion biopsies	378 (59.4%)
	Systematic biopsies	255 (40.1%)
	Missing	3 (0.5%)
Total number of cores		13 [10, 16]
Prostate Cancer		
	No Pca	267 (42%)
	ISUP 1	77 (12.1%)
	ISUP 2	90 (14.2%)
	ISUP 3	64 (10.1%)
	ISUP 4	73 (11.5%)
	ISUP 5 Missing	59 (9.3%) 6 (0.9%)

Results are shown as median, lower and upper quartiles. Number cases. Missing data. ccm - cubic centimeters. PSA– Prostate-specific Antigen

**Table 2:** Prostate Cancer detection in systematic biopsies

	*n*/*N*	(%)
**ISUP score**		
0	127/255	49.8%
1	18/255	7.1%
2	16/255	6.3%
3	16/255	6.3%
4	33/255	12.9%
5	42/255	16.5%
Missing	3/255	1.2%

ISUP grade group - International Society of Urological Pathology

**Table 3:** Prostate Cancer detection in MRI fusion biopsies

	*n*/*N*	(%)
**ISUP score**		
0	140/378	37%
1	59/378	15.6%
2	74/378	19.6%
3	48/378	12.7%
4	40/378	10.6%
5	17/378	4.5%
Missing	0/378	0%

ISUP grade group - International Society of Urological Pathology

A Spearman’s rank correlation was conducted to assess the relationship between PIRADS score and ISUP grade in the fusion biopsies. The analysis revealed a statistically significant positive correlation (ρ = 0.467, *p* < 0.001), suggesting that higher PIRADS scores are associated with higher ISUP grades (Table 4).

**Table 4:**
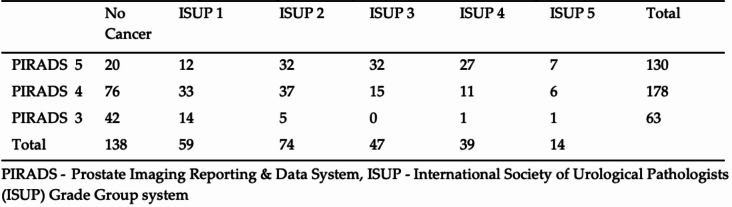
PIRADS lesion and prostate biopsy histologically results (in fusion biopsy only)

In total, we recorded 12 complications, for a complication rate of 1.9%. Details of Clavien Dindo grade are described in Table 5. We found 1.1% (7/636) infectious complications needing antibiotic treatment (see Table 6). Three patients diagnosed with urosepsis were evaluated based on systemic inflammatory response syndrome (SIRS) parameters (see Table 7). All patients presented with elevated body temperature (≥ 39.8 °C) and leukocytosis (> 12 × 10⁹/L). Heart rates varied, with two patients exceeding 90 bpm, while one remained below this threshold. Respiratory rate data were unavailable. Blood cultures confirmed *Escherichia coli* (*E. coli*) as the causative pathogen in all cases. Two patients were treated with intravenous antibiotics on a normal ward and showed clinical improvement. However, one patient, presenting with the highest leukocyte count (30.9 × 10⁹/L) and a heart rate of 96 bpm, required intensive care unit (ICU) admission due to the severity of their condition.

**Table 5:** All complications

	*n*/*N*	(%)
**Complications in total**	12/636	1.90%
Clavien Dindo		
Grade 1	0/636	
Grade 2	11/636	1.70%
Grade 3	0/636	
Grade 4	1/636	0.16%
Grade 5	0/636	

**Table 6:** Infectious complications

	*n*/*N*	(%)
**Infectious complications**	7/636	1.10%
Type of infectious complication		
Prostatitis	3/636	0.50%
Epididymitis	1/636	0.16%
Urosepsis requiring hospitalization	2/636	0.30%
Urosepsis with ICU admission	1/636	0.16%

**Table 7:** Clinical SIRS-Parameters of patient with Urosepsis

	Temperature (°C)	Heart Rate (/min.)	Respiratory Rate (per min.)	White blood cell count ( 10⁹/L)	Postitve Blood Cluture	
Pat. 1	39,9	92	n.a.	14,3	E.coli	Hospitalization
Pat. 2	39,8	66	n.a.	21,7	E.coli	Hospitalization
Pat. 3	40	96	n.a.	30,9	E.coli	Hospitalization with ICU

SIRS– Systemic Inflammatory Response Syndrome; n.a.– not available; ICU– Intensive Care Unit;

Four patients reported gross hematuria requiring bladder irrigation. One patient needed catheterization due to urinary retention. For the 11 patients who were excluded from data analysis, no complications could have been identified in chart review.

A patient is diagnosed with SIRS if they meet at least two of the following criteria:

- **Temperature**: A temperature above 100.4 °F (38 °C) or below 96.8 °F (36 °C).
- **Heart rate**: A heart rate above 90 beats per minute.
- **Respiratory rate**: A respiratory rate above 20 breaths per minute, or a partial pressure of carbon dioxide below 32 mm Hg.
- **White blood cell count**: A white blood cell count above 12 × 10⁹/L or below 4 × 10⁹/L; or more than 10% immature forms.

We could not determine any risk factors for infectious complications (age *p* = 0.44, diabetes mellitus *p* = 0.23, indwelling catheter *p* = 0.26, number of cores *p* = 0.6, prostate volume *p* = 0.44), post-interventional bleeding (age *p* = 0.75, under anticoagulant agent *p* = 0.5, number of cores *p* = 0.14, prostate volume *p* = 0.10), PSA level < 20 vs. > 20 ng/ml *p* = 0.12). Table 7 highlights those patients who suffered from an infectious complication and their clinical details (see Table 8).

**Table 8:** Clinical parameters of patients suffering from an infectious complication

Age	Previous biopsy	Previous prostatitis	Diabetes mellitus	Anti-coagulant	Catheter	Previous surgery	PSA	Prostate volume	Number of cores	Fusion biopsy	ISUP	Fever	Complication
72	no	no	yes	no	no		7.0	40.0	10	no	5	yes	Prostatitis
57	> 1y	no	no	no	no		17.0	115.0	16	yes	0	yes	Prostatitis
65	no	no	no	no	yes		28.3	77.0	10	no	0	yes	Urosepsis
64	> 1y	no	no	no	no		11.4	72.0	20	yes	0	yes	Epididymitis
67	no	no	no	ASS	no		4.0	39.0	16	yes	2	yes	Urosepsis
79	no	no	no	ASS	no	TUR-P	63.0	25.0	10	no	4	yes	Urosepsis with ICU
87	no	yes	yes	ASS	no	TUR-P	38.8	10.0	15	yes	5	yes	Prostatitis

Age in years, Diabetes mellitus (Type I and II), Number of biopsy cores, ISUP - International Society of Urological Pathologists (ISUP) Grade Group system, ICU- Intensive Care Unit

## Discussion

### Main findings

Our study reported on complications after transperineal prostate biopsy without peri-interventional antibiotic prophylaxis and without routine screening for bacteriuria in a single-center retrospective cohort of 636 patients.

Most trials do not mention if patients were excluded from the trial after performing urine analysis showing bacteriuria. In our cohort, patients were not systematically screened for underlying UTI/bacteriuria if they did not report signs or symptoms for a UTI. As the transperineal route does not systematically injure the rectal mucosa, there is little rationale for systematic screening of the rectal flora.

Furthermore, we did not exclude patients with a “high risk for infectious complication after prostate biopsy.” High risk for infectious complication after prostate biopsy is not clearly defined. In the NORAPP trial [8], the following criteria were defined for high risk: indwelling urinary catheter, immunodeficiencies, high risk of infective endocarditis, or a history of thromboembolic disease.

As mentioned above, in our daily clinical practice we only check for an underlying UTI with urine analysis before performing a prostate biopsy if obvious signs or symptoms for a significant UTI as fewer, painful voiding abnormalities or visible pyuria with an indwelling catheter are apparent. If so, the scheduled biopsy is postponed, with further diagnostics, including urine microscopy and cultures, guiding treatment. In our cohort of 636 consecutive patients, there were no obvious signs or symptoms for a significant UTI and consequently no urine analysis was performed. As a result, there was no need to postpone any prostate biopsy due to an asymptomatic UTI. While postponing the biopsy remains an option in cases of confirmed infection, we did not encounter such a situation in our study.

We reported a complication rate of 1.9% with 11 Clavien Dindo grade (CD) 2 complications and one CD 4 complication. The most common complications were infectious complications. The rate of infectious complications for the transperineal prostate biopsy without antibiotic prophylaxis in our cohort (about 1%) is much lower than what has generally been reported for transrectal prostate biopsy with antibiotic prophylaxis (approx. 5–7%) [10]. Notably, three of the infectious complications in our cohort were cases of sepsis, as defined by the SIRS criteria. The post-biopsy sepsis rate is a key factor driving the shift among urologists toward the transperineal approach. It must be emphasized that post-biopsy infectious complications are not necessarily a surrogate for post-biopsy sepsis.

### Findings of biopsy results

Histologically confirmed prostate cancer was more often found in fusion biopsy than in systematic biopsies (Tables 2 and 3). High-grade prostate cancer (ISUP > 3) was more frequently found in the group who underwent systematic biopsies. An explanation could be that we did not perform upfront MRIs when there was a high risk for prostate cancer, such as PSA > 20 ng/ml or suspicious DRE. Therefore, systematic biopsies had a negative selection bias for ISUP grade compared to the group of MRI fusion biopsies.

### Limitations

The main limitation of this study is that follow-up data has been collected with a retrospective chart review. The total number of low-grade complications may have been a little higher, as not all patients may have been referred back to our clinic nor do they all remember if they had been prescribed an oral medication (i.e. antibiotics) by a private consultant. Higher-grade complications (Clavien Dindo Grade > 2) were likely not missed, as our tertiary medical care center comprises the only urological unit in a radius of about 75 km. Risk factors for infectious or bleeding complications did not meet statistical significance. Indeed 2 out of the 3 patients with an occurring urosepsis after the procedure had a pre biopsy PSA level over 20ng/ml (28.3 and 63.0ng/ml). This might lead to the estimation to screen those patients with very high PSA levels routinely on UTIs with pre-interventional urine cultures to avoid complications although this paraemter reached no significance in our study. This may have been due to our cohort’s relatively low number of complications. Our cohort with 650 patients and less than 2% complications may be insufficient to provide a correct insight into the prevalence of complications. A larger cohort may be necessary when analyzing complications and their risk factors with low incidence.

## Conclusions

Our report confirmed existing data showing a low number of infectious complications when using the transperineal route for prostate biopsies despite the omission of antibiotic prophylaxis and despite the omission of systematic screening for underlying bacteriuria. Omitting antibiotic prophylaxis and omitting screening for bacteriuria seems to be safe in transperineal prostate biopsies.

## Data Availability

No datasets were generated or analysed during the current study.
